# A Rare Case of Pancreatic Adenocarcinoma With Incidental Synchronous Hepatic and Sigmoid Colonic Metastasis: A Case Report and Literature Review

**DOI:** 10.7759/cureus.23921

**Published:** 2022-04-07

**Authors:** Mohammed N AlAli, Mohammed Ayesh, Amany Fathaddin, Hamad AlQahtani

**Affiliations:** 1 General Surgery, King Khalid University Hospital, King Saud University, Riyadh, SAU; 2 Radiology, King Khalid University Hospital, King Saud University, Riyadh, SAU; 3 Pathology, King Saud University Medical City, Riyadh, SAU; 4 Surgery/Hepatibiliary, King Khalid University Hospital, King Saud University, Riyadh, SAU

**Keywords:** cancer, tumor, mass, sigmoid colon metastasis, liver metastasis, pancreatic adenocarcinoma

## Abstract

Metastasis of pancreatic adenocarcinoma to the colon is a very rare condition that might be underdiagnosed and underreported in the literature. We report a very rare case of incidental findings of sigmoid metastasis secondary to pancreatic adenocarcinoma in a 60-year-old Saudi male, who is a non-smoker with a normal medical and surgical history. The patient presented to a primary care clinic with abdominal bloating and vague on/off abdominal pain for almost 1 year as well as unintentional weight loss without lower gastrointestinal (GI) symptoms. After the case was discussed in the multidisciplinary tumor board, the patient was started on systematic palliative chemotherapy. However, after receiving the first cycle, he started to deteriorate rapidly and succumbed to secondary cardiopulmonary arrest. Cases of synchronous metastasis of pancreatic adenocarcinoma to the colon might not be well known or common. However, a high index of suspicion and individualizing the workup tools may need to be used.

## Introduction

The worldwide age-standardized incidence rate (ASIR) of pancreatic adenocarcinoma is low (4.8/100,000 with a slight male predominance) but slowly increasing with a 5-year overall survival rate of about 6% [1]. The ASIR of Saudi Arabia was reported at 2.26 and 1.41 per 100,000 for males and females, respectively, with mortality at only 2.2/100,000, which is lower than the worldwide incidence [1]. It has an extremely poor prognosis as it is usually associated with a late diagnosis. However, only around 20% have resectable disease at the time of diagnosis. It metastasizes to the liver, lungs, regional lymph nodes, or peritoneum, but very rarely to the colon or the sigmoid [2-4].

To the best of our knowledge, only a few cases of synchronous or metachronous colonic metastasis secondary to pancreatic cancer were reported in the literature [2,5]. Therefore, we report a rare and very interesting case of incidental synchronous pancreatic adenocarcinoma with concurrent hepatic and sigmoid colonic metastasis.

## Case presentation

**Figure 1:**
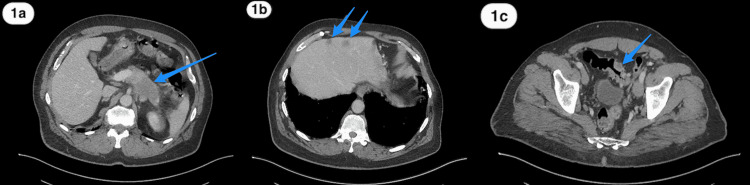
Abdomen CT scan (a) A contrast-enhanced abdomen CT scan axial image showing an ill-defined non-enhancing low-density solid mass in the pancreatic body and tail (blue arrow). (b) A contrast-enhanced abdomen CT scan axial image showing liver metastases (blue arrows). (c) A contrast-enhanced pelvis CT scan axial image showing focal non-uniform wall thickening of the sigmoid colon (blue arrow). CT, computed tomography.

**Figure 2:**
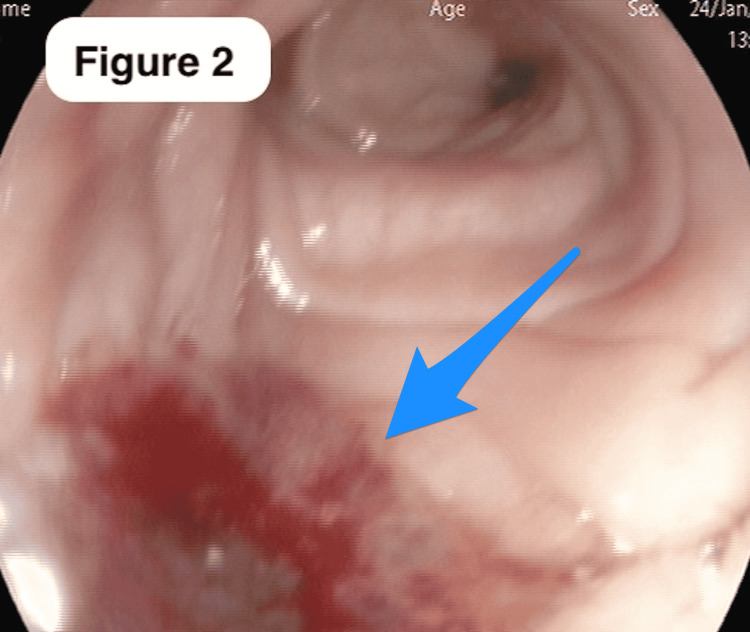
Colonoscopy A colonoscopic view of the sigmoid colon showing a significant finding of an erythematous flat mucosa about 5 x 3 cm, which was found to be a metastatic pancreatic adenocarcinoma.

**Figure 3:**
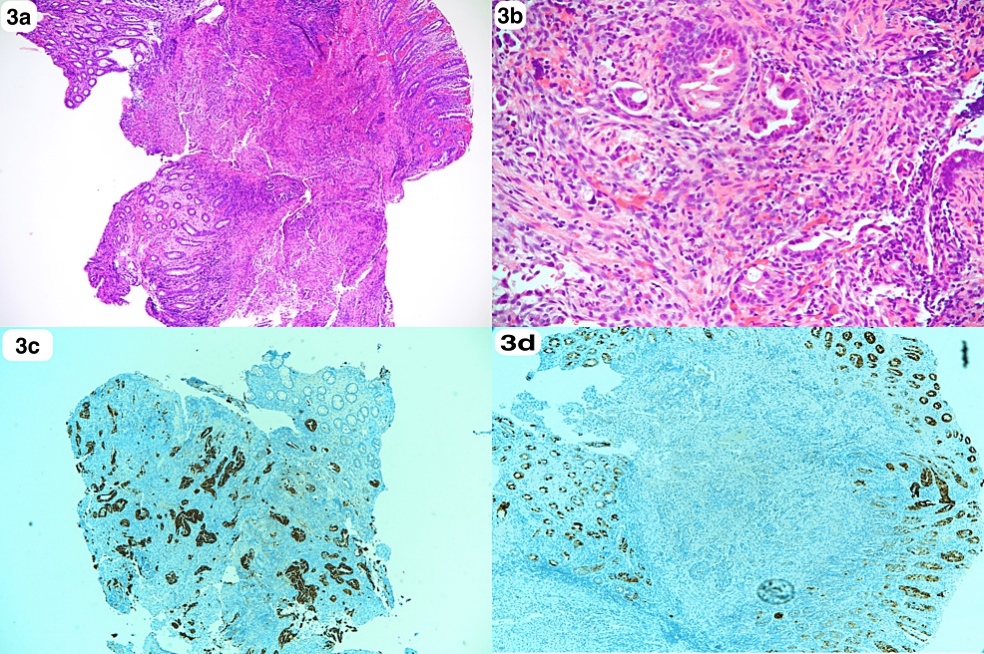
Histopathology of colonic lesion (a, b) Sigmoid colon biopsy: colonic mucosa with underlying neoplastic, muscle infiltrative glands (H&E x4, x20). (c) Immunohistochemical stains showing a positive reaction to CK 7 in the infiltrative glands, while colonic mucosa is negative (CK 7 x 4). (d) Immunohistochemical stains showing a negative reaction to CK 20 in the infiltrative glands (CK20 x4).

**Figure 4:**
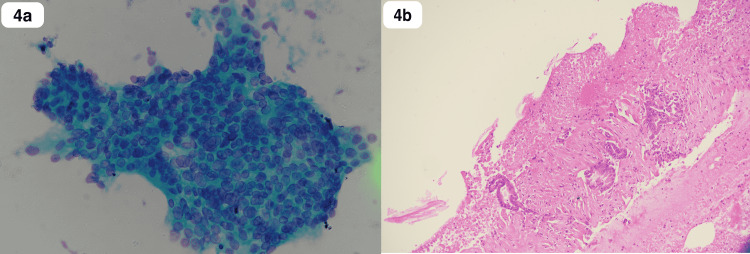
Histopathology of pancreatic body lesion (a) EUS-guided FNA: crowded and disoriented cells with variation in size, nuclear enlargement, and irregular nuclear membranes (PAP stain x40). (b) Cell block preparation: desmoplastic reaction around neoplastic glands (H&E stain x10). EUS, endoscopic ultrasound; FNA, fine-needle aspiration; PAP, Papanicolaou; H&E, hematoxylin and eosin.

A 60-year-old Saudi male, a non-smoker with a normal medical, surgical, and family history, presented for the first time to our primary care clinic with abdominal bloating and vague on/off left hypogastric abdominal pain for almost 1 year with no previous history of seeking medical advice. He reported an unintentional weight loss of 20 kg over the previous 3 months. There were no other upper or lower gastrointestinal (GI) symptoms, no alcohol or drug abuse, and no history of irritable bowel syndrome or inflammation. On examination, the patient looked comfortable, not in pain, distress, or jaundice, and vitally stable. The abdomen examination was normal. Laboratory works up including Hgb (162.0 g/L), leukocytes (3.9 x 109/L), liver enzymes, coagulation profile, renal profile (76 mcmol/L), C-reactive protein, and alkaline phosphatase, amylase, lipase, Hgb A1c (6.2%), triglycerides (1.38 mmol/L), and calcium, all were normal. Initially, an abdominal ultrasound revealed two worrisome hypoechoic focal lesions noted at the right hepatic lobe, the largest measuring 1.8 x 1.8 cm and the other 1.5 x 1.1 cm. Therefore, the patient was referred to the gastroenterology unit for proper assessment and management. An enhanced triphasic CT abdomen was done, which showed a hypodense pancreatic lesion and two hypodense hepatic lesions, initially reported as probably representing neoplastic lesions like metastatic pancreatic adenocarcinoma or lymphoma (Figure 1a-c). Also, colonoscopy was done as a part of the initial workup, which showed a significant finding of erythematous flat mucosa about 5 x 3 cm in size at the sigmoid colon which was unlikely to be a primary colonic lesion (Figure 2). A biopsy was also taken in addition to multiple large diverticula. The biopsy was reported as moderately differentiated adenocarcinoma in favor of metastasis (Figure 3a-d). Also, ultrasound-guided fine-needle aspiration cytology of pancreatic body lesion was performed, which reported moderately differentiated adenocarcinoma of pancreatic ductal origin as well (Figure 4a-b).

The patient was then referred to the hepatobiliary surgery clinic. Liver biopsy under ultrasound guidance was requested and reported as metastatic adenocarcinoma of pancreatobiliary origin. Also, the following tumor markers were reported: carbohydrate antigen 19-9 of 24,208.1 kU/l, carcinoembryonic antigen 17.3 ug/L, and alfa-fetoprotein 4.20 KIU/L. Therefore, the case was discussed in the multidisciplinary tumor board and planned for systematic palliative chemotherapy. Unfortunately, after receiving the first cycle of Folfirinox, the patient entered an acute confessional state but there was no proven stroke. He was also diagnosed to have myocarditis. Then he started to deteriorate rapidly as he was intubated and shifted to the intensive care unit where he succumbed to secondary cardiopulmonary arrest.

## Discussion

It is well known that metastatic dissemination of pancreatic cancer is commonly extending to the liver, lungs, regional lymph nodes, and peritoneum. However, in this study, we focus on a very interesting phenomenon about the metastasis of adenocarcinoma of the pancreas to the colon, specifically to the sigmoid, which might change the workup for pancreatic adenocarcinoma soon. Over the last few decades, there was no significant change in the long-term relative survival rate (28.2% in the first year and 6.9% in 5 years) in pancreatic cancer [1,2,4,6].

There is a paucity of cases in the literature about a rare entity of metastatic pancreatic cancer to the colon, especially similar to our case of the incidental finding of metastasis to the sigmoid colon in the absence of lower GI symptoms [2].

Most pancreatic adenocarcinoma cases are discovered without a typical presentation, and metastases are discovered postoperatively [4]. In our case, the patient presented with abdominal bloating and vague on/off, left hypogastric abdominal pain, as well as unintentional weight loss. However, there was no colonic obstruction, change in bowel habits, or PR bleeding, which might indicate the presence of colorectal metastasis, though being discovered preoperatively as a synchronous tumor [4,6].

During workup, a CT scan is not always able to detect small colonic abnormality unlike colonoscopy, which is not routinely used as a part of the workup for pancreatic cancer [5]. In our case, a screening colonoscopy found an atypical lesion for primary colonic adenocarcinoma and a biopsy confirmed that it was a metastatic lesion.

Till date, the only curative intent in the management of pancreatic cancer is surgical resection, though only a small portion of the pancreatic cancer population will benefit from it (about 80% had the locally advanced or metastatic disease), which is the case in our patient as he was started on a palliative regimen of systemic chemotherapy for controlling disease spread [4]. In addition to this, most recurrence cases from post-surgical resection and adjuvant chemotherapy are reported as metastasis disease, which might be secondary to chemotherapy regimen [7] or underdiagnosed synchronous pancreatic colorectal metastasis [4].

## Conclusions

Since unexpected metastases of pancreatic adenocarcinoma to the colon are being reported in recent years as either an underdiagnosed or an underreported entity, a high index of suspicion and individualizing as well as tailoring proper workup tools may need to be used. Therefore, further studies and reports are encouraged.
